# Genetic variation implicates plasma angiopoietin-2 in the development of acute kidney injury sub-phenotypes

**DOI:** 10.1186/s12882-020-01935-1

**Published:** 2020-07-17

**Authors:** Pavan K. Bhatraju, Max Cohen, Ryan J. Nagao, Eric D. Morrell, Susanna Kosamo, Xin-Ya Chai, Robin Nance, Victoria Dmyterko, Joseph Delaney, Jason D. Christie, Kathleen D. Liu, Carmen Mikacenic, Sina A. Gharib, W. Conrad Liles, Ying Zheng, David C. Christiani, Jonathan Himmelfarb, Mark M. Wurfel

**Affiliations:** 1 Division of Pulmonary, Critical Care and Sleep Medicine, Department of Medicine, University of Washington, 325 9th Avenue, Seattle, WA 98104 USA; 2 Kidney Research Institute, Division of Nephrology, Department of Medicine, University of Washington, Seattle, USA; 3 Department of Bioengineering, University of Washington and Center for Cardiovascular Biology, Seattle, USA; 4 Institute of Stem Cell and Regenerative Medicine, University of Washington, Seattle, USA; 5 Department of Epidemiology, University of Washington, Seattle, USA; 6 Division of Pulmonary, Allergy, and Critical Care and Center for Clinical Epidemiology and Biostatistics, Department of Medicine, University of Pennsylvania, Philadelphia, USA; 7 Divisions of Nephrology and Critical Care Medicine, University of California San Francisco, San Francisco, USA; 8 Department of Medicine, University of Washington, Seattle, USA; 9 Departments of Environmental Health and Epidemiology, Harvard TH Chan School of Public Health, Harvard University and Pulmonary and Critical Care Division, Cambridge, USA; 10 Department of Medicine, MA General Hospital/Harvard Medical School, Boston, USA

**Keywords:** Acute kidney injury, Genetics, Endothelium

## Abstract

**Background:**

We previously identified two acute kidney injury (AKI) sub-phenotypes (AKI-SP1 and AKI-SP2) with different risk of poor clinical outcomes and response to vasopressor therapy. Plasma biomarkers of endothelial dysfunction (tumor necrosis factor receptor-1, angiopoietin-1 and 2) differentiated the AKI sub-phenotypes. However, it is unknown whether these biomarkers are simply markers or causal mediators in the development of AKI sub-phenotypes.

**Methods:**

We tested for associations between single-nucleotide polymorphisms within the *Angiopoietin-1, Angiopoietin-2,* and *Tumor Necrosis Factor Receptor 1A* genes and AKI- SP2 in 421 critically ill subjects of European ancestry. Top performing single-nucleotide polymorphisms (FDR < 0.05) were tested for cis-biomarker expression and whether genetic risk for AKI-SP2 is mediated through circulating biomarkers. We also completed in vitro studies using human kidney microvascular endothelial cells. Finally, we calculated the renal clearance of plasma biomarkers using 20 different timed urine collections.

**Results:**

A genetic variant, rs2920656C > T, near *ANGPT2* was associated with reduced risk of AKI-SP2 (odds ratio, 0.45; 95% CI, 0.31–0.66; adjusted FDR = 0.003) and decreased plasma angiopoietin-2 (*p* = 0.002). Causal inference analysis showed that for each minor allele (T) the risk of developing AKI-SP2 decreases by 16%. Plasma angiopoietin-2 mediated 41.5% of the rs2920656 related risk for AKI-SP2. Human kidney microvascular endothelial cells carrying the T allele of rs2920656 produced numerically lower levels of angiopoietin-2 although this was not statistically significant (*p* = 0.07). Finally, analyses demonstrated that angiopoietin-2 is minimally renally cleared in critically ill subjects.

**Conclusion:**

Genetic mediation analysis provides supportive evidence that angiopoietin-2 plays a causal role in risk for AKI-SP2.

## Background

Acute kidney injury (AKI) affects 40–60% of patients admitted to the intensive care unit (ICU) [1] and contributes to poor short- and long-term outcomes [2–4]. Genetic studies to date have focused on associations between genetic variants and the risk for AKI comparing cases (AKI) to controls (no AKI) [5, 6]. However, this framework may be limited because cases of AKI are highly heterogeneous with different precipitants and biological profiles [7]. Combining such AKI patients to maximize sample size may result in dilution of genetic statistical signals that might only be present in one pathophysiologically distinct subset of the AKI population. Another limitation is that AKI in critically ill populations is often a complication of serious insult, such as sepsis, surgery, shock, pneumonia, and trauma. The use of controls without the development of AKI can be problematic. Controls carrying a high-risk genetic variant might not develop AKI if they do not also experience a similar acute insult as cases, and thus would be classified as non-cases, attenuating any potential association signal [8]. The use of biologically distinct AKI sub-phenotypes in genetic association studies overcomes prior limitations in phenotyping AKI by specifically focusing on the AKI population and by comparing two biologically distinct sub-phenotypes [9].

We recently identified two AKI sub-phenotypes (AKI-SP1 and AKI-SP2) applying latent class analysis methodology to a panel of 29 clinical and biomarker variables in two independent critically ill AKI populations [10]. Notably, AKI-SP2 was associated with worse hospital outcomes (e.g. mortality, new dialysis and 7-day renal non-recovery) compared to AKI-SP1. We next identified these AKI sub-phenotypes in a previously completed multi-center randomized control trial, Vasopressin versus Norepinephrine Infusion in Patients with Septic Shock (VASST) [11]. The VASST trial studied whether the choice of vasopressor therapy improved mortality in subjects with septic shock. While the AKI population in the clinical trial had no difference in mortality to vasopressor therapy, AKI-SP1 had a mortality benefit with vasopressin compared to AKI-SP2 having no mortality difference. To our knowledge, this is the first example of identifying treatment responsive AKI sub-groups in the critically ill.

Notably, no single variable was statistically better than the other variables to identify AKI-SP2 (Table S1). In contrast, a three-variable model, using plasma angiopoietin-2 (ANG-2), angiopoietin-1 (ANG-1), and soluble tumor necrosis factor receptor-1 (sTNFR-1), had the optimal predictive performance to differentiate AKI sub-phenotypes (C-statistic 0.93). Lower ANG-2, lower sTNFR-1 and higher ANG-1 were associated with lower risk of AKI-SP2. Studies in animal models of AKI have shown that these plasma biomarkers are involved in the pathophysiology and severity of AKI [12–16]. However, it is unknown whether these plasma biomarkers play a causal role in the development of clinical AKI sub-phenotypes. The identification of causal markers could inform targets for drug development to prevent or treat the development of AKI in the critically ill and could assist in patient risk-stratification.

Genetic mediation analysis is one of several causal inference approaches that can identify the potential mechanism by which an independent variable (e.g., genetic variant) affects the outcome (e.g., AKI sub-phenotypes) via an explanatory mediator (e.g., biomarker of endothelial dysfunction). This approach has been widely applied in clinical data to understand causal mechanisms of disease [17–19]. We hypothesized that cis-quantitative trait loci (QTLs) in the *ANGPT1, ANGPT2, and TNFRSF1A* genes influence the development of AKI sub-phenotypes by regulating circulating levels of their respective biomarkers (ANG-1, ANG-2 or sTNFR-1).

## Methods

### Study populations

We previously reported the identification of AKI sub-phenotypes using a prospectively collected ICU cohort: identification of Single Nucleotide Polymorphisms (SNPs) Predisposing to Altered Acute Lung Injury Risk (iSPAAR) [10, 20]. The iSPAAR population is a genome-wide case-control study of risk of acute respiratory distress syndrome (ARDS) that included patients with and without ARDS. The iSPAAR population included subjects from previously completed randomized control trials and from a prospectively enrolled ICU cohort. Details of the study design for each population enrolled in iSPAAR have been previously described; Albuterol for the Treatment of Acute Lung Injury (ALTA) [21], Fluid and Catheter Treatment Trial (FACTT) [22], Enteral omega-3 fatty acid, gamma-linolenic acid, and antioxidant supplementation in acute lung injury (OMEGA) [23] and Molecular Epidemiology of Acute Respiratory Distress (MEA) at the Massachusetts General Hospital [24]. Within iSPAAR, study enrollment occurred 48 h after ICU admission. At study enrollment DNA and plasma were collected for genotyping and biomarker analysis followed by AKI ascertainment. AKI was defined as an increase in serum creatinine (SCr) of ≥0.3 mg/dl or 50% from “baseline” SCr. The baseline SCr was defined as the lowest value prior to study enrollment [25–27]. AKI was also defined using a modified urine output criteria (daily output instead of every 6 h).

To determine renal clearance of plasma biomarkers, we enrolled a prospective cohort in the Harborview medical and surgical intensive care units, Critical Illness AKI Cohort (CIA). Subjects were eligible for enrollment if they met 2 of 4 systemic inflammatory response syndrome criteria, had a clinically-suspected infection, and had an indwelling urinary catheter in place. A timed urine collection was completed that lasted at least 2–4 h and EDTA plasma samples were collected at the beginning and the end of the timed urine collection. Clearance was calculated using the formula Clearance *(X)* = *U(X)* * *V/ P(X)*, where *U(X)* represents the urine concentration of solute *X*, *V* indicates the urine volume over the 2–4-h collection period, and *P (X)* represents the average plasma concentrations of solute *X* from the initial and final blood collections.

### Genotyping strategy

Genotyping was performed in 421 patients of European ancestry using the Illumina 660 platform (Illumina, San Diego, CA). Genotyped data was quality controlled using a sample call rate filter > 0.97, minor allele frequency (MAF) > 0.01 and SNP call rate > 0.95. After quality control, 238 SNPs ±50 kilobases of *ANGPT1, ANGPT2* and *TNFRSF1A* were found. After linkage disequilibrium (LD) pruning of a r^2^ of 0.8, 48 SNPs were removed leading to a total of 190 SNPs used in association tests. Imputation was conducted using the 1000 Genomes Project reference panel using IMPUTE2 v2.3.0 [28, 29].

### Plasma protein assessment

Plasma and urinary biomarkers were measured using electrochemiluminescent immunoassays (Meso Scale Discovery (MSD), Rockville, MD), as previously described [10]. The blood was collected in EDTA-treated sterile tubes, urine was collected in sterile containers and both were centrifuged immediately. Plasma and urine was then aliquoted and frozen at − 80 °C. The samples were stored for different durations but they were thawed in a single batch and only once for running the biomarker measurements for this study. All biomarker measurements were performed in duplicates at Harborview Pulmonary Research Laboratories.

### In Silico analyses

To test SNPs for expression of QTL effects, we queried the Genotype-Tissue Expression (GTEx) Portal [30].

### Cell culture

Human kidney microvascular endothelial cells (HKMECs) were purified from fetal kidneys after voluntary pregnancy interruptions between 100 and 135 days postconception. Informed consents for the use of fetal tissues were obtained from patients. We then randomly chose 9 different donor HKMECs, thawed and plated half a million cells in T25 flasks coated with 0.2% gelatin and maintained in EBM-2 basal medium containing 1% antibiotic-antimycotic (Life Technologies), 10% FBS, 100 μg/mL ECGS, 50 μg/mL Heparin, and 20 ng/mL VEGF (R&D), for 48 h till confluency. At 48 h, we purified genomic DNA from the HKMECs and cell supernatants were collected. We successfully genotyped cells from 8 donors.

### Statistical analysis

Patient demographic variables are reported as either mean +/−standard deviation or as median and quartiles. First, we used logistic regression to test for an association between the 190 SNPs and the development of AKI-SP2 compared to AKI-SP1 using an additive genetic model (Golden Helix, MT). Our model was adjusted for the following covariates: age, sex, sepsis and first five principal components. The Eigenstrat method v4.2 was used to calculate the principal components and the top five were included as covariates. Odds ratios (ORs) are reported with 95% confidence intervals. For the analysis between genetic variants and AKI sub-phenotypes, we corrected for multiple comparisons by using a Benjamini-Hochberg false discovery rate (FDR) threshold < 0.10, which estimates that less than 10% of the associations with an FDR value at or below this level are false positives [31]. In a sensitivity analysis, an imputed genotype was used to identify additional SNPs associated with AKI-SP2.

Second, we used linear regression adjusting for age, sex and sepsis to determine associations between top performing SNPs and log_2_ transformed biomarker concentrations. Third, we completed causal inference analysis to test the association between genetic variants and AKI-SP2 and the potential mediation of the association by plasma biomarker concentrations. The mediation analysis was performed using the non-linear implementation of structural equation modeling implemented in the mediation package for STATA [32, 33]. Additional details of the causal inference analysis are provided in the online supplement. Fourth, we determined associations between genetic variants and AKI severity, measured by maximum serum creatinine, via logistic regression. Additional details of materials and methods are provided in the online supplement. In the analysis, we evaluated 190 SNPs and used a conservative *p-value* of 0.05/190 = 2.6 × 10^− 4^. Given that approximately 40% of ICU patients develop AKI, and an expected control (AKI-SP1) to case (AKI-SP2) ratio of 1.5, an expected sample size of 421, and a MAF of at least 0.30, we will have 81% power to detect a relative risk of 1.5 or greater [34]. Analyses were completed using STATA (Version 15) and Goldenhelix (Version 4.0). All studies were approved by the Human Subjects Division at the University of Washington. Written informed consent was obtained from all subjects enrolled.

## Results

### Characteristics of populations

Of the 425 patients from the validation cohort in our previous work, 421 had genotyping data available. Demographics and baseline clinical characteristics are described in Table 1. All subjects were of European ancestry. A total of 267 (63%) were classified as AKI-SP1 and 154 (37%) as AKI-SP2. Subjects who developed AKI-SP2 had higher illness severity on presentation (mean acute physiology and chronic health evaluation (APACHE) III scores, 111 ± 26 vs 74 ± 24), were more likely to have sepsis (84% vs 66%) and were more likely to be treated with vasopressors (79% vs 42%) compared to AKI-SP1.

**Table 1:** Demographic and clinical data

Participant Characteristics	AKI-SP1 (***N*** = 267)	AKI-SP2 (***N*** = 154)	***p-value***
**Baseline Demographics**			
Age (year)	57 ± 18	56 ± 17	0.84
Male (%)	164 (61)	104 (68)	0.19
Body Mass Index (kg/m^2^)	29 ± 8	29 ± 8	0.97
Race (%)			
Caucasian	267 (100)	154 (100)	1.00
**Co-Morbidities**			
Diabetes Mellitus	72 (27)	40 (26)	0.69
Cirrhosis	6 (2)	14 (9)	< 0.01
**ICU Events**^a^			
APACHE III Scores	74 ± 24	111 ± 26	< 0.01
Sepsis – 3	178 (67)	132 (86)	< 0.01
Vasopressors	113 (42)	124 (81)	< 0.01
24 h urine output (ml)	1680 (1140–2665)	1199 (563–2050)	< 0.01
**ICU Laboratory Values**^a^			
Maximum White Blood Cell Count (10*9)	16 ± 8	17 ± 13	0.25
Low Hematocrit (%)	30 ± 6	31 ± 6	0.74
Low Sodium (mEq/L)	137 ± 6	135 ± 5	< 0.01
Low Albumin (g/dL)	2.4 ± 0.6	2.2 ± 0.7	< 0.01
Low Platelets (10^9^/L)	184 ± 101	85 ± 75	< 0.01
Low Sodium Bicarbonate (mEq/L)	22 ± 5	17 ± 5	< 0.01
**Biomarker Concentrations (pg/ml)**			
Angiopoietin-2	23,458 (12,208-38,707)	74,972 (48,294-128,421)	< 0.01
Angiopoietin-1	2361 (1090-5102)	778 (398–1998)	< 0.01
Soluble Tumor Necrosis Factor Receptor-1	10,581 (6828-15,742)	25,815 (16,084-36,211)	< 0.01
**Outcomes**			
Length of ICU stay, d	7.5 ± 7.3	8.3 ± 8.7	< 0.01
Maximum 7 day Serum Creatinine (mg/dL)	1.6 (1.2–2.3)	2.7 (1.8–4.3)	< 0.01
28-day mortality	36 (14)	57 (37)	< 0.01

Data shown as mean ± standard deviation, n (%), median (interquartile range), as appropriate. ^a^All ICU Events and ICU laboratory values are the maximum or minimum value at the time of study enrollment

### Genetic Variation Near *ANGPT2,* rs2920656, is Associated with AKI-SP2

**Fig. 1:**
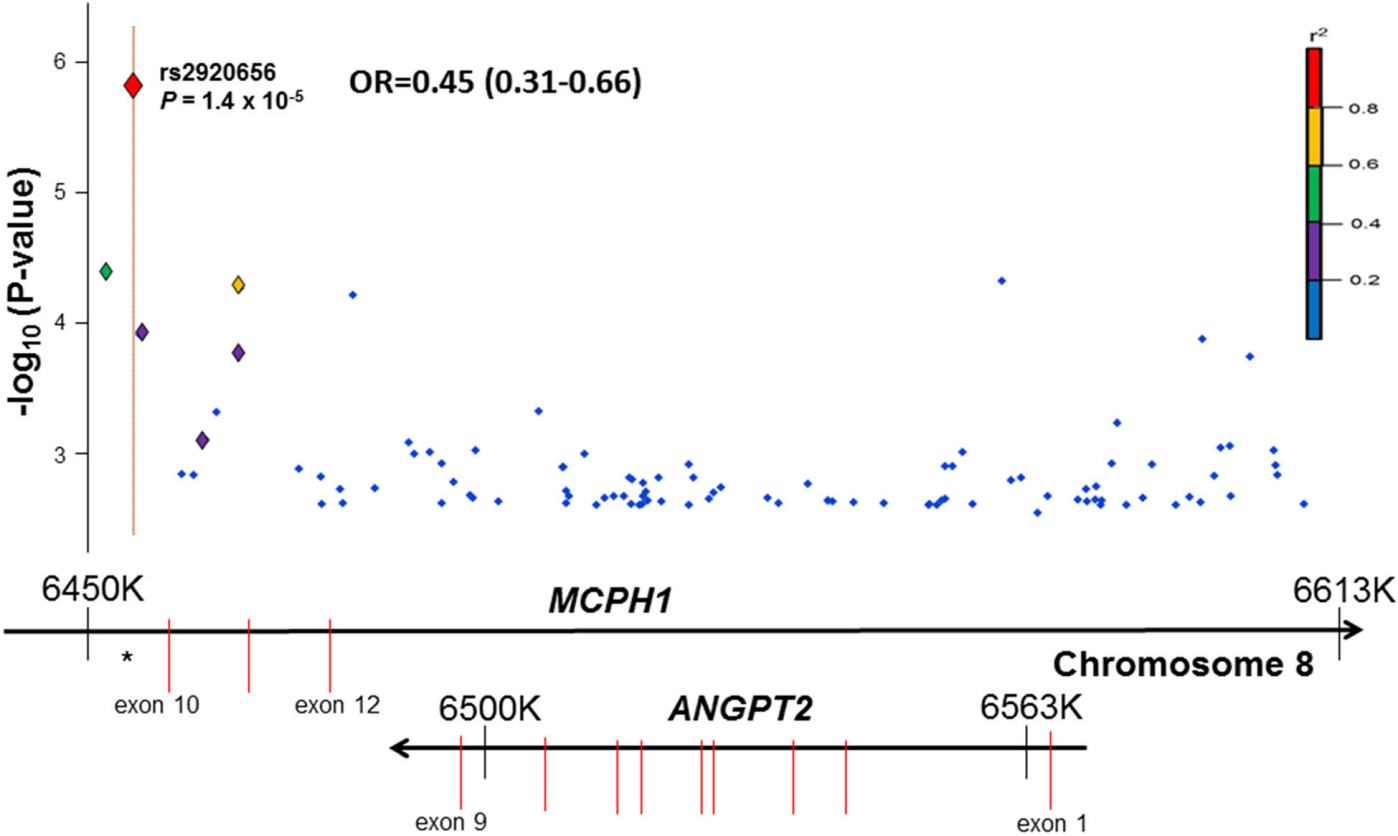
Regional association plot of *ANGPT2* region with acute kidney injury sub-phenotype 2 (AKI-SP2). Results are plotted as genomic locus versus –log (*P* value) for the association with AKI-SP2. The *P* value reflects an additive genetic model for association with AKI-SP2. Each locus is also annotated with the background genome recombination rate. Underneath the regional association plot is a schematic of the *ANGPT2* and *MCPH1* genes with exons represented as vertical red lines. The top single nucleotide polymorphism (SNP) was rs2920656. Plot was generated using Goldenhelix based on RefSeq Genes 105v2 in the CEU population, a Utah population of European ancestry

Of the 190 SNPs ±50 kilobases of the genes, 72 were near *ANGPT1*, 100 were near *ANGPT2* and 18 near *TNFRSF1A* gene. We identified one SNP meeting an FDR < 0.05 that was associated with AKI-SP2 compared to AKI-SP1 (Table 2 and Fig. 1). No significant associations were observed with SNPs in or near *ANGPT1* or *TNFRSF1A* (Table S2 and S3).

**Table 2:** SNPs Most Associated with AKI-SP2

SNP	Associated Gene	Chromosome: Coordinate	Function	Minor Allele Frequency (1000 Genomes)	Odds Ratio (95% CI)^**a**^	***P-value***	FDR Corrected ***P-value***
1. rs2920656	MCPH1	Chr8:6329510	Intron	0.284	0.45 (0.31–0.66)	1.38 × 10^−5^	0.0026
2. rs2442473	ANGPT2 MCPH1	Chr8:6358293	Intron	0.082	0.40 (0.22–0.74)	0.001902	0.0723
3. rs2920689	MCPH1	Chr8:6343499	Intron	0.310	0.59 (0.42–0.82)	0.001538	0.0731
4. rs2959779	MCPH1	Chr8:6443380	Intron	0.055	0.27 (0.11–0.67)	0.001307	0.0828
5. rs2440399	MCPH1	Chr8:6325975	Intron	0.207	0.52 (0.34–0.78)	0.000984	0.0935

*Definition of abbreviations*: *SNP* single nucleotide polymorphism, *CI* confidence interval, *FDR* False Discovery Rate

^a^Additive genetic model adjusted for age, gender, sepsis and 5 principal components

The SNP demonstrating the strongest association with risk for AKI-SP2 was rs2920656 (OR, 0.45; 95% CI, 0.31–0.66; *p* < 1.4 × 10^− 5^; FDR = 0.003). This intronic SNP is ≈ 30 kb downstream to the 3′ position of *ANGPT2* and explained approximately 3% of the variance in development of AKI-SP2 (R^2^). Because only a small number of subjects were homozygous rs2920656 (*n* = 26), we also tested associations between rs2920656 and AKI-SP2 in a dominant genetic model, which gave consistent results (OR, 0.42; 95% CI, 0.28–0.64; *p* < 1.4 × 10^− 6^). We also tested for additional, potentially stronger, associations within the *ANGPT2* locus using imputed genotypes but did not find any associations stronger than that observed with rs2920656 (Table S4).

In a sensitivity analysis we tested whether the inclusion of critically ill patients without AKI would influence the genetic association. We grouped patients with no AKI and AKI-SP1 together and determined whether rs2920656 was still strongly associated with a decreased risk of AKI-SP2. In this analysis, 839 patients had either no AKI or AKI-SP1 and 154 had AKI-SP2. The risk of developing AKI-SP2 again was significantly reduced with having at least one T allele for rs2920656 (OR 0.31; 95% CI, 0.19–0.52; *p* < 0.001) (Table S5). In another sensitivity analysis, we tested if rs2920656 was associated with decreased risk of AKI-SP2 within each of the four different studies that were included in iSPAAR. Within each of the three randomized control trials (ALTA, FACTT and OMEGA) and the ICU prospective cohort (MEA), the point estimate was consistent with the minor allele of rs2920656 demonstrating a decreased risk for the development of AKI-SP2 (Table S6). Thus, rs2920656 was analyzed further to determine the association with plasma biomarker concentrations.

### T allele of rs2920656 is associated with decreased plasma ANG-2

**Fig. 2:**
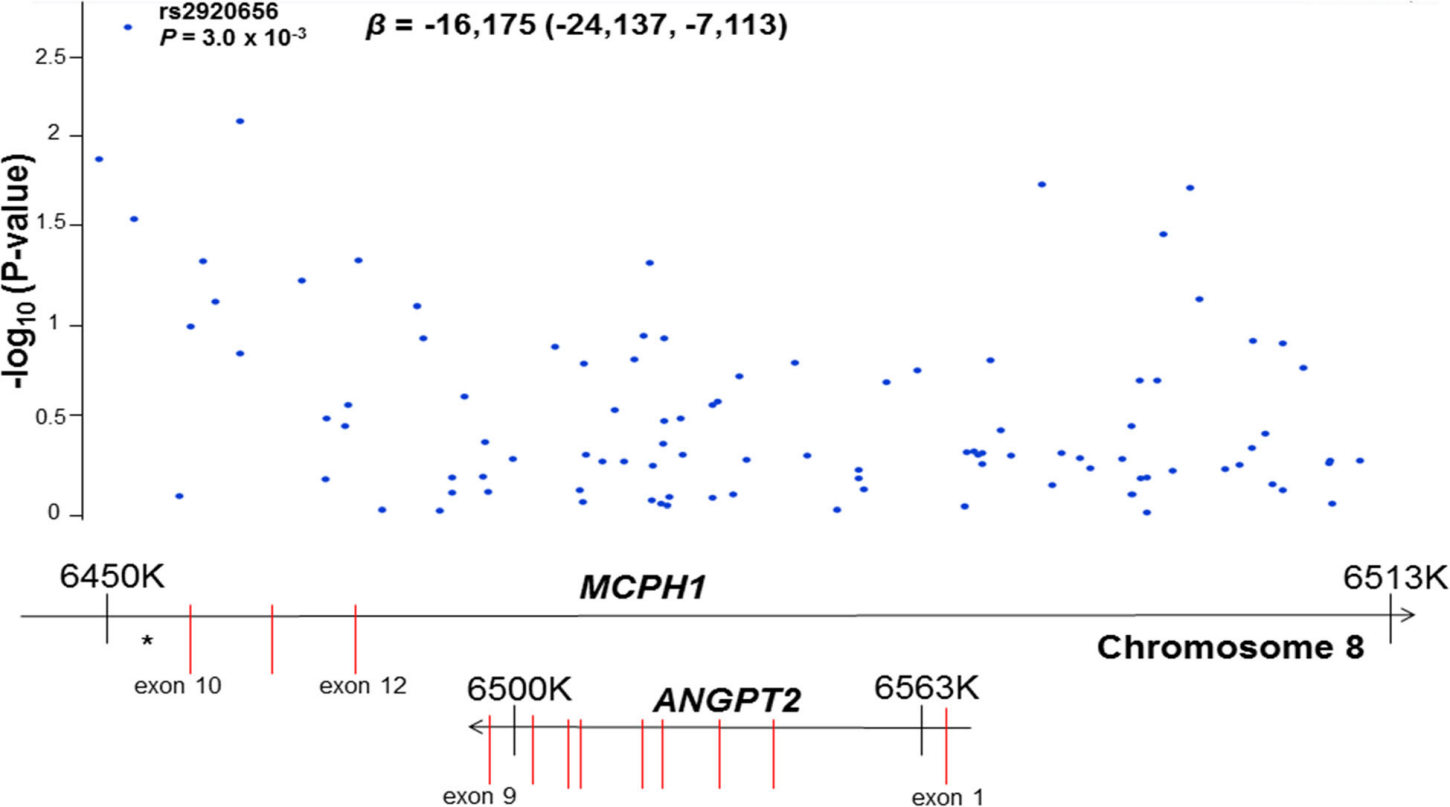
Regional association plot of *ANGPT2* region with plasma Angiopoietin 2 (ANG-2) concentrations (ng/ml). Results are plotted as genomic locus versus –log (*P* value) for the association with ANG-2 concentrations. The top variant was rs2920656 which was the top variant for our previous association of genotypes on AKI-SP2. Underneath the regional association plot is a schematic of the *ANGPT2* and *MCPH1* genes with exons represented as vertical red lines. Plot was generated using Goldenhelix based on RefSeq Genes 105v2 in the CEU population, a Utah population of European ancestry

We next analyzed the association between rs2920656 and plasma ANG-2 concentrations. Adjusting for age, gender and sepsis each copy of the T allele of rs2920656 was associated with decreased log_2_ plasma ANG-2 concentrations (*β* = − 0.09; 95% CI -0.15, − 0.04; *P* = 0.002). Subjects homozygous for the C allele showed the highest concentrations of plasma ANG-2 (40,683 pg/ml; interquartile range (IQR) 19,374-73,205), while subjects homozygous for the T allele showed the lowest plasma ANG-2 concentrations (28,308 pg/ml (IQR 14,340-42,944). In addition, of the 100 SNPs tested near the *ANGPT2* gene region, rs29206565 was the most strongly associated with plasma ANG-2 concentrations (Fig. 2).

### Mediation analysis suggests ANG-2 is causal in the development of AKI-SP2

**Fig. 3:**
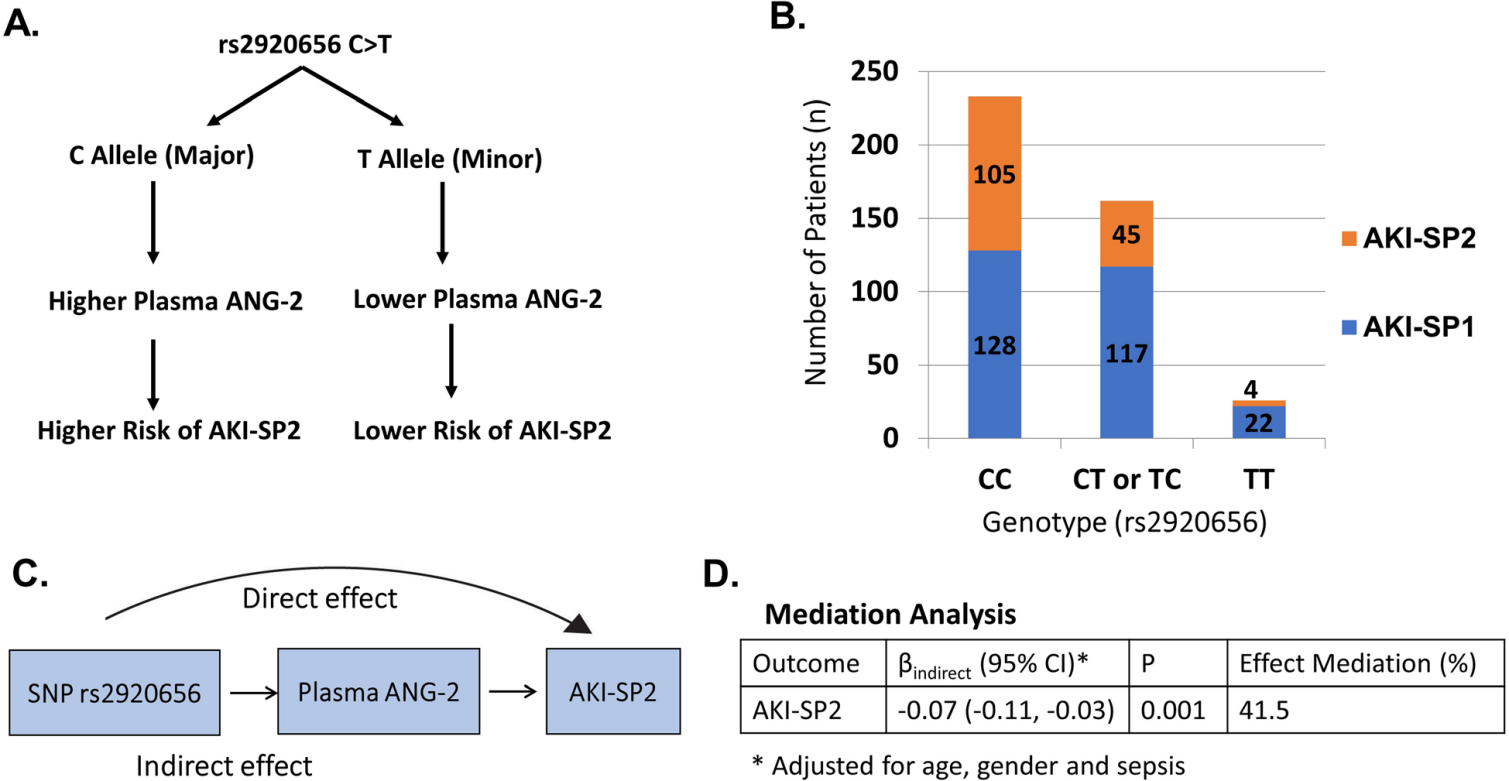
Association between single-nucleotide polymorphism (SNP) rs2920656 and AKI sub-phenotypes and the effects mediated through plasma angiopoietin-2 concentrations. **a** Schematic of rs2920656, concentrations of plasma ANG-2 and risk of AKI-SP2. **b** Number of patients with AKI-SP2 by SNP. **c** The mediation model. **d** Results of mediation analyses for AKI-SP2. Results are described as indirect and total prognostic effect of the SNP that was mediated through ANG-2 plasma concentrations, 95% CI, *p*-value and the proportion of the effected mediated

We tested for evidence that the association between rs2920656 and risk for AKI-SP2 is mediated through plasma ANG-2 concentrations (Fig. 3). The total effect of rs2920656 on AKI-SP2 was *β*_total_ = − 0.16 per allele (95% CI -0.24, − 0.10, *p* = 1.0 × 10^− 4^), which suggests that for each minor allele (T allele) of the genetic variant the risk of developing AKI-SP2 decreases by 16%. Causal mediation analysis detected a significant indirect effect for rs2920656 on AKI-SP2 that was mediated through plasma ANG-2 concentrations (*β*_indirect_, − 0.07 per allele; 95% CI -0.11, − 0.03; *p* = 0.001), which means the proportion of effect between rs2920656 and AKI-SP2 that is mediated by ANG-2 concentrations is 41.5%.

### T allele of rs2920656 is associated with decreased AKI severity at 7 days

Next, we tested the association of rs2920656 with traditional criteria for AKI severity, such as maximum serum creatinine and increase in serum creatinine within 7 days after study enrollment. In a dominant genetic model, the T allele of rs2920656 was associated with a decrease in serum creatinine of − 0.44 mg/dL (95% CI, − 0.77, − 0.10), *p* = 0.01) after adjusting for age, gender, body mass index and sepsis status. The minor allele of rs2920656 was also associated with a decrease in the rise in serum creatinine between baseline and day 7 (*β =* − 0.25, 95% CI, − 0.46, − 0.03; *p =* 0.03*).*

### T allele of rs2920656 is associated with lower ANG-2 in cell culture

**Fig. 4:**
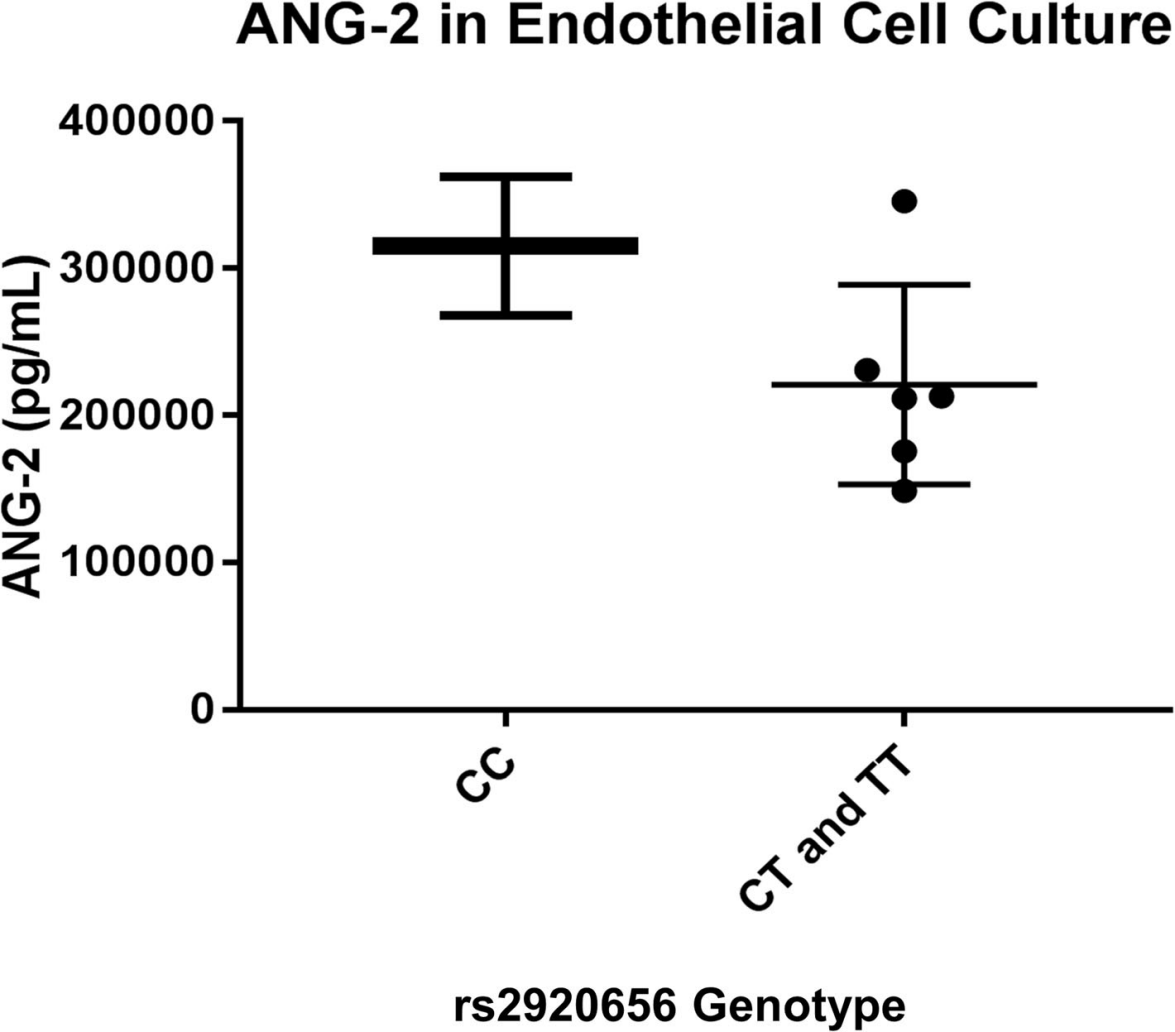
ANG-2 concentrations by rs2920656 genotype in human microvascular kidney endothelial cells (HKMECs). Of 8 human kidney samples, 2 were CC, 5 were CT and 1 was TT for rs2920656. The T allele of rs2920656 was associated with decreased ANG-2 concentrations. Student t-test was used combining the CT and TT genotypes and generated a *p*-value = 0.07 for the difference between genotypes and ANG-2 concentrations. The mean ANG-2 concentrations are 315,000 pg/mL for CC, 220,900 pg/mL for CT and TT

We conducted in vitro experiments and in silico analyses to determine the functional significance of rs2920656. Of 8 different human fetal kidney tissue samples, 2 were CC, 5 were CT and 1 was TT for rs2920656. In a dominant genetic model, ANG-2 concentrations were numerically greatest in endothelial cells from donors homozygous for the C allele and lower in carriers of the T allele, *p* = 0.07 (Fig. 4). In the GTEx project database, rs2920656 was not associated with *ANGPT2* gene expression. However, two other SNPs (rs41311412 and rs2515591) that are in moderate LD (r^2^ = 0.23 and D’ = 0.86) with rs2920656 were associated with reduced *ANGPT2* gene expression (*p* = 4.1 × 10^− 5^) in tibial artery, a tissue which is highly enriched for endothelial cells (Table S7).

### Plasma ANG-2 is minimally cleared by the kidneys

To determine whether differences in kidney function could influence plasma ANG-2 concentrations, we measured ANG-2 renal clearance in critically ill subjects with and without AKI. In 20 different timed urine sample collections with bookended plasma samples, the median serum creatinine was 0.86 mg/dL with an interquartile range (IQR) 0.69 to 1.45 mg/dL. The median plasma ANG-2 concentration was 10,261 pg/mL (IQR 6210–19,115 pg/mL). In contrast, urinary ANG-2 concentrations were 50-fold lower with a median of 206 pg/mL (IQR 11–839 pg/mL). The calculated renal clearance of ANG-2 was < 1 mL/min for all timed urine collections, suggesting that plasma ANG-2 concentrations are not increased simply as a function of worsening AKI (Table 3).

**Table 3:** ANG-2 Renal Clearance in Subjects with and without AKI

Timed Urine Collection	Average Serum Creatinine (mg/dL)	Average Plasma ANG-2 (pg/mL)	Urine ANG2 (pg/mL)	ANG-2 Renal Clearance (mL/min)
1	3.24	29,312.89	4671.87	0.216
2	3.52	27,136.51	5915.63	0.353
3	0.82	5061.54	10.00	0.002
4	0.89	4782.57	553.81	0.161
5	0.61	4435.69	169.09	0.064
6	0.53	3867.01	10.00	0.006
7	0.75	10,426.38	11.80	0.004
8	0.69	9699.06	14.58	0.002
9	0.60	6593.72	10.00	0.004
10	0.82	8350.90	190.68	0.010
11	0.71	15,886.06	220.09	0.003
12	0.69	10,096.66	243.74	0.018
13	2.57	26,288.02	2097.31	0.079
14	2.24	24,769.31	1967.59	0.165
15	2.16	22,259.65	1696.53	0.074
16	0.99	10,839.17	10.00	0.002
17	0.44	2624.28	10.00	0.007
18	1.20	18,067.95	192.79	0.014
19	1.14	13,683.26	527.93	0.069
20	1.13	8829.19	245.35	0.095

Serum creatinine and plasma ANG-2 calculated based on the average measurement from the plasma sample collected at the beginning and at the end of the time urine collection. Clearance calculated using the formula Clearance *(X)* = *U(X)* * *V/ P(X)*, where *U(X)* represents the urine concentration of solute *X*, *V* indicates the urine volume over the 2–4-h collection period, and *P (X)* represents the average plasma concentrations of solute *X* from the initial and final blood collection

## Discussion

The search for susceptibility genes in AKI has been hindered by heterogeneity within the clinical disease phenotype. Using AKI sub-phenotypes, we have discovered that a genetic variant near the *ANGPT2* gene, rs2920656, is protective against the development of AKI-SP2. Causal inference analysis suggests that plasma ANG-2 concentrations mediated 41.5% of the genetic association between rs2920656 and AKI-SP2 risk in subjects of European ancestry. Furthermore, in-vitro experiments demonstrated that the minor allele of rs2920656 may lead to lower ANG-2 protein release by human kidney endothelial cells. In addition, plasma ANG-2 is minimally renally cleared, suggesting that elevated plasma ANG-2 concentrations are unlikely to result from kidney injury. Overall, these findings provide evidence that plasma ANG-2 plays a mechanistic role in the host’s response to critical illness leading to AKI-SP2. Efforts to target the Ang-Tie2 axis may limit AKI severity and resulting poor clinical outcomes [35].

The *ANPT2* gene is 100% nested within the microcephalin (*MCPH1*) gene. Mutations in the *MCPH1* gene have been associated with diseases of neurogenesis and renal cell carcinoma [36]. We have shown that rs2920656 is an intronic variant in *MCPH1* that regulates plasma ANG-2 concentrations. Previous studies have identified genetic variants in *MCPH1* [37] and *ANGPT2* [38] that are associated with ANG-2 concentrations. It is also conceivable that rs2920656 influences *MCPH1* expression. However, to our knowledge no role for *MCPH1* in acute or chronic kidney disease or vascular injury has been described. In contrast, multiple pre-clinical and clinical studies have demonstrated the important role of plasma ANG-2 in the development of AKI [16, 39, 40].

Multiple reports have implicated plasma biomarkers of endothelial function in the pathophysiology of AKI [39, 41, 42]. ANG-1 and -2 are vascular endothelial growth factors that both bind to the endothelial tyrosine kinase receptor (Tie-2) but have context dependent activities [43]. ANG-1 is released by pericytes and platelets and is an agonist for the Tie-2 receptor. ANG-1 is protective by stabilizing the endothelium and preventing microcirculatory capillary leakage, a hallmark of AKI [44]. In contrast, ANG-2 typically acts as an antagonist to the Tie-2 receptor and promotes endothelial permeability [45] and inflammation [46, 47]. The *ANGPT2* gene encodes for circulating ANG-2, which is released from endothelial cells during an inflammatory stimulus. Animal studies have shown that inhibition of ANG-2 binding, augmenting ANG-1 concentrations [41, 44], or activation of Tie-2 [46] decreases endothelial leak and protects against AKI. Taken together these studies implicate a mechanistic role of the ANG-Tie2 axis in AKI. Here we demonstrate genomic regulation of plasma ANG-2 as another piece supporting the causative role of ANG-2 in the development of a severe form of AKI, AKI-SP2.

The strong association between plasma ANG-2 and development of AKI-SP2, raises the question of whether ANG-2 is similar to creatinine: filtered by the kidney and elevated levels are simply reflections of decreased renal filtration. To demonstrate that plasma ANG-2 concentrations are not simply a marker but causal in the development of severe AKI, we provide two lines of evidence. First, using genetic causal inference analysis, we have shown that genetic variation near the *ANGPT2* gene is associated with ANG-2 plasma concentrations and the development of AKI-SP2. Second, unlike creatinine (113 Da), ANG-2 (57,000 Da) is a large molecule that is unlikely to be regularly filtered at the glomerulus. In a critically ill population, we have demonstrated minimal renal clearance of ANG-2. Thus, elevations in plasma ANG-2 concentrations are unlikely to be due to differences in renal filtration and, instead, may be involved in the pathophysiology of AKI in critically ill patients.

It is important to note that individual genetic variants likely have small overall effects on disease development because AKI is likely a polygenic disease. The strength of this analysis is identification of a genetic variant that supports ANG-2 as causal in the development of AKI. Even variants with modest effect sizes provide opportunities for the investigation of potential novel causal pathways using genetic medication analysis. For example, cardiovascular disease, similar to AKI, is a polygenic trait with many genetic variants each explaining a small proportion of the risk. Regardless, three SNPs that explained only 0.4 to 2% of the variance in c-reactive protein levels allowed the determination that c-reactive protein was not causal in the development of ischemic vascular disease [48], and these findings were confirmed in subsequent studies [49].

Our work has several strengths. First, we used AKI sub-phenotypes to leverage precision in the phenotype definition and to maximize sample size to discover genetic variants. Second, causal inference analysis suggests that 41.5% of rs2920656-associated risk for developing AKI-SP2 is explained by plasma ANG-2 levels. This provides clinical evidence, to build on work from animal studies, that modulation of plasma ANG-2 concentrations may improve outcomes in critical illness associated AKI. Third, to account for potential residual confounding and to link Ang-2 production specifically to kidney endothelial cells, we completed in-vitro experiments using HKMECs. Fourth, using a unique ICU cohort with timed urine collection samples and before and after plasma samples, we were able to demonstrate that minimal amounts of plasma ANG-2 is filtered by the kidney. Thus, the strong association of ANG-2 with kidney specific outcomes is likely not confounded by issues of reverse causation.

Our study has limitations. Our sample size was relatively small. However, our well-defined quantitative trait (AKI-SP2) allowed us to identify a genetic association with the limited number of critically ill patients with AKI. Second, analyses were limited to patients of European ancestry in order to reduce genetic admixture, maximize power and because of differences in allelic frequencies among ethnic backgrounds. Future work is warranted to study alternative ethnic populations to determine if similar genomic variation influences plasma ANG-2 concentrations. Third, while there was a trend in lower ANG-2 measurements in PTECs with the minor allele, the results were not statistically significant. However, the human samples are difficult to obtain and even with a small sample size we saw a consistent direction. Fourth, due to the uniqueness of this dataset we were unable to find a similar patient population to replicate our findings. Our dataset included well-phenotyped patients with AKI, with genomic, plasma and clinical outcome data. However, within the four individual populations included in iSPAAR there was a consistent direction in effect between rs2920656 and development of AKI-SP2. Future work is warranted to understand the influence of rs2920656 on sub-phenotype development and AKI specific clinical outcomes.

## Conclusion

In summary, we identified a genetic variant near the *ANGPT2* gene that is associated with plasma ANG-2 concentrations and the development of AKI-SP2 among a critically ill population. We also tested this association through studies completed in HKMECs. Our findings suggest that plasma ANG-2 plays a causal role in the development of AKI-SP2 and believe efforts to target the Ang-Tie2 axis may prevent the development of poor clinical outcomes.

## Supplementary information

**Additional file 1: Supplement Data File.** Includes supplement tables referenced in the manuscript.

## Data Availability

The datasets generated during and/or analyzed during the current study are available in the dbGAP genotypes and phenotypes repository, accession number: phs000631.v1.p1 and web link is https://www.ncbi.nlm.nih.gov/projects/gap/cgi-bin/study.cgi?study_id=phs000631.v1.p1

## Abbreviations

AKI: Acute kidney injury
AKI-SP1: Acute kidney injury sub-phenotype 1
AKI-SP2: Acute kidney injury sub-phenotype 2
ANG-1: Angiopoietin 1
ANG-2: Angiopoietin 2
*ANGPT1*: Angiopoietin-1 gene
*ANGPT2*: Angiopoietin-2 gene
APACHE III: Acute Physiology Age Chronic Health Evaluation
CI: Confidence Interval
ICU: Intensive Care Unit
KDIGO: Kidney Disease Improving Global Outcomes
MAF: Minor allele frequency
SNP: Single-nucleotide polymorphism
sTNFR-1: Soluble Tumor Necrosis Factor Receptor – 1
*TNFRSF1A*: *Tumor necrosis factor receptor surface-1A*
