# Supplementary material for: Genetic variation implicates plasma angiopoietin-2 in the development of acute kidney injury sub-phenotypes
Source: BMC Nephrol. 2020 Jul 17;21:284. doi: 10.1186/s12882-020-01935-1 (PMC7368773; doi:10.1186/s12882-020-01935-1)
Supplement: Supplementary file 1 — Additional file 1: Supplement Data File. Includes supplement tables referenced in the manuscript. [file 12882_2020_1935_MOESM1_ESM.docx]

**Online Supplementary Material**

**Title:** Genetic variation implicates plasma angiopoietin-2 in the development of acute kidney injury sub-phenotypes

**Authors:** Pavan K. Bhatraju, MD. MSc; Max Cohen MD. PhD; Ryan Nagao PhD; et al.

**Table of Contents**

Methods

Table S1. Top 10 Variables to Predict AKI-SP2 membership

Table S2. SNPs Most Associated with AKI-SP2 in *ANGPT1* Gene

Table S3. SNPs Most Associated with AKI-SP2 in *TNFRSF1A* Gene

Table S4. Top Performing SNPs from Imputed Dataset using 1000 genomes

Table S5. Risk of developing AKI-SP2 continues to be lower even when including the no AKI population.

Table S6. Genetic Association of rs2920656 and AKI-SP2 within iSPAAR populations

Table S7. GTEx database gene expression in tibial artery tissue for rs41311412 and rs2515591

**Methods for Mediation Analysis**

The mediation analysis was performed using the non-linear implementation of the structural equation modeling implemented in the mediation package for STATA. Plasma ANG-2 was log-converted to account for the right skewed distribution of the measurements. The STATA package allowed us to test how robust our estimates of mediated and direct effects were to unmeasured confounders and issues of measurement error. The linear structural equation models were conditioned on age, gender and sepsis status. Robust methods were used to estimate all confidence intervals for the linear structural equation models.

**Plasma Protein Measurements**

Blood samples were diluted to fit within the dynamic range of each assay. Samples were measured in singlets and samples that fell below the lower limit of detection or above the upper limit of detection were assigned the value of the lowest standard or the highest standard multiplied by the dilution factor, respectively. As an additional quality control measure, we freeze/thawed a random subset of these samples and re-measured all analytes. The replication results were highly consistent with average Pearson Correlation for all assays at 0.95 with a standard deviation of 0.06 (data not shown). Biomarker concentrations were log _2_ transformed before analysis given a right skewed distribution. The CV in control samples were low 3.6%.

**Cell Culture**

Human kidney microvascular endothelial cells (HKMECs) were purified from fetal kidneys after voluntary pregnancy interruptions between 100 and 135 days postconception in compliance with the Institutional Review Board protocol at the University of Washington. Informed consents for the use of fetal tissues were obtained from patients. The fetal kidneys at that stage contained established nephrons including glomeruli, tubules and interstitium, and have begun to produce urine. HKMECs were isolated through initial depletion of epithelial cells and supplementing with vascular endothelial growth factor in culture media. Isolated HKMECs were VE Cadherin +/CD31+ CD45- PDGFR*B*-*(47)*. We then randomly chose 9 different donor HKMECs, thawed and plated half a million cells in T25 flasks coated with 0.2% gelatin and maintained in EBM-2 basal medium containing 1% antibiotic-antimycotic (Life Technologies), 10% FBS, 100 μg/mL ECGS, 50 μg/mL Heparin, and 20 ng/mL VEGF (R&D), for 48 hours till confluency. At 48 hours, we purified genomic DNA from the HKMECs and cell supernatants were collected. We genotyped two SNPs, rs2920656 and rs2979656, using commercially available TaqMan assays and run on the ABI PRISM Sequence Detection System (SDS; Applied Biosystems, Foster City, CA) as previously described*(48)*. We successfully genotyped cells from 8 donors. Genotyping of one donor was unsuccessful. In another donor, we were only able to genotype rs2979656 and then inferred the allele for rs2920656 from the allele at rs2979656 based on the high LD between these variants (r^2^=0.9 and D’=1).

**Table S1. Top 10 Variables to Predict AKI-SP2 membership**

| **Clinical Variables and Biomarkers** | **C-statistic^†^**  **(95%CI)** |
| --- | --- |
| 1. ANG-2/ANG-1 + sTNFR-1 | 0.93 (0.91 – 0.95) |
| 2. ANG-2/ANG-1 | 0.87 (0.84 – 0.90) |
| 3. ANG-2 | 0.87 (0.84 – 0.90) |
| 4. sTNFR-1 | 0.85 (0.81 – 0.89) |
| 5. IL-8 | - 1. 0.80 – 0.87) |
| 6. Platelet Count | 0.80 (0.76 – 0.84) |
| 7. IL-6 | - 1. (0.74 – 0.84) |
| 8. Sodium Bicarbonate | - 1. 0.71 – 0.80) |
| 9. ANG-1 | - 1. 0.68-0.78) |
| 10. Vasopressor Use | 0.68 (0.64 – 0.73) |

**Table S2. SNPs Most Associated with AKI-SP2 near *ANGPT1* Gene**

| **SNP** | **Position** | **Function** | **1000 genomes MAF** | **Odds Ratio**  **(95% CI)** | **P-value** | **FDR Corrected P-value** |
| --- | --- | --- | --- | --- | --- | --- |
| rs10505101 | Chr 8: 108321414 | Intron | 0.316 | 0.56 (0.37 – 0.85) | 0.00013 | 0.1388 |
| rs6982586 | Chr 8: 108353121 | Intron | 0.179 | 0.67 (0.47-0.96) | 0.0004 | 0.3875 |
| rs2436557 | Chr 8: 108376234 | Intron | 0.251 | 1.5 (1.0 – 2.1) | 0.0006 | 0.3982 |
| rs2584363 | Chr 8: 108458296 | Intron | 0.159 | 0.73 (0.53 – 1.00) | 0.0007 | 0.4021 |
| rs10505101 | Chr 8: 108227559 | Intron | 0.316 | 1.5 (1.05 – 2.27) | 0.0005 | 0.4059 |

Odds ratio adjusted for age, gender, sepsis and 5 principal components. MAF – minor allele frequency, CI confidence interval, FDR- False Discovery Rate. Additive model

**Table S3. SNPs Most Associated with AKI-SP2 near *TNFRSF1A* Gene**

| **SNP** | **Position** | **Function** | **1000 genomes MAF** | **Odds Ratio**  **(95% CI)** | **P-value** | **FDR Corrected P-value** |
| --- | --- | --- | --- | --- | --- | --- |
| rs3136551 | Chr12:6424121 | Intron | 0.060 | 1.70 (0.72 – 3.60) | 0.0018 | 0.82 |
| rs11064213 | Chr12:6450637 | Intron | 0.343 | 0.84 (0.56 – 1.23) | 0.0023 | 0.89 |
| rs10774435 | Chr12:6486381 | Intron | 0.193 | 1.16 (0.81 – 1.65) | 0.0024 | 0.92 |
| rs1001127 | Chr12:6472004 | Intron | 0.208 | 1.13 (0.81 – 1.56) | 0.0024 | 0.94 |
| rs10849451 | Chr12:6394940 | Intron | 0.385 | 1.04 (0.78 – 1.38) | 0.0026 | 0.95 |

Odds ratio adjusted for age, gender, sepsis and 5 principal components. MAF – minor allele frequency, CI confidence interval, FDR- False Discovery Rate. Additive model

**Table S4. Top Performing SNPs from Imputed Dataset using 1000 genomes**

| **SNP** | **Gene** | **Position** | **Function** | **1000 genomes MAF** | **P-value** | **r**^2^ | **D’** |
| --- | --- | --- | --- | --- | --- | --- | --- |
| 1. rs2920656 | MCPH1 | Chr8:6329510 | Intron | 0.284 | 1.38x10^-5^ | Ref | Ref |
| 1. rs2442492 | MCPH1 | Chr8:6329561 | Intron | 0.4519 | 0.000699452 | 0.5525 | 0.8957 |
| 1. rs2515587 | MCPH1 | Chr8:6330026 | Intron | 0.4529 | 0.000699452 | 0.5525 | 0.8957 |
| 1. rs2442490 | MCPH1 | Chr8:6330806 | Intron | 0.4535 | 0.000699452 | 0.5525 | 0.8957 |
| 1. rs2442489 | MCPH1 | Chr8:6330881 | Intron | 0.4545 | 0.000699452 | 0.5525 | 0.8957 |
| 1. rs2979656 | MCPH1 | Chr8:6331758 | Intron | 0.4856 | 0.000743947 | 0.9031 | 1.0 |
| 1. rs536403150 | MCPH1 | Chr8:6326168 | Intron | 0.3113 | 0.001144757 | 0.4313 | 0.7914 |
| 1. rs2440399 | MCPH1 | Chr8:6325975 | Intron | 0.4541 | 0.001221387 | 0.5525 | 0.8957 |
| 1. rs2442496 | MCPH1 | Chr8:6327274 | Intron | 0.4513 | 0.001221387 | 0.5525 | 0.8957 |
| 1. rs1550690 | MCPH1 | Chr8:6338937 | Intron | 0.3115 | 0.00125218 | 0.7198 | 0.8927 |

Top 10 SNPs are all in LD based on r2 or D’ with rs2920656.

**Table S5. Risk of developing AKI-SP2 continues to be lower even when including the no AKI population.**

| **AKI Sub-groups** | **N (%)** | **rs2920656 genotype (CC)** | **rs2920656 genotype (CT and TT)** | **Odds Ratio (95% CI)*** |
| --- | --- | --- | --- | --- |
| No AKI + AKI-SP1 | 839 (84) | 583 (81) | 256 (92) |  |
| AKI-SP2 | 157 (16) | 136 (19) | 21 (8) | 0.31 (95% CI 0.19 -0.52) |

*Adjusted for Age, gender, body mass index and sepsis-3 status

**Table S6. Genetic Association of rs2920656 and AKI-SP2 within iSPAAR populations**

|  | **Number of Centers** | **Type of Study** | **Total number of patients included in Replication Group n (%)** | **OR (95% CI), *p-value***** |
| --- | --- | --- | --- | --- |
| Molecular Epidemiology of Acute Respiratory Distress (MEA) at the Massachusetts General Hospital | 1 | Prospective Observational | 184 (44) | 0.56 (0.29 – 1.08), *p=0.083* |
| Albuterol for the Treatment of ALI (ALTA)* | 33 | Randomized Control | 71 (17) | 0.24 (0.08 – 0.70), *p=0.009* |
| Fluid and Catheter Treatment Trial (FACTT)* | 20 | Randomized Control | 103 (26) | 0.31 (0.13 – 0.74), *p=0.008* |
| Enteral omega-3 fatty acid, gamma-linolenic acid, and antioxidant supplementation in acute lung injury (OMEGA)* | 44 | Randomized Control | 63 (15) | 0.64 (0.26 – 1.57), *p=0.333* |

*NHLBI ARDS Network Clinical Trials

******Adjusted for age, gender, body mass index and sepsis-3 status

**Table S7. GTEx database gene expression in tibial artery tissue for rs41311412 and rs2515591**
